# Familial writer’s cramp: a clinical clue for inherited coenzyme Q_10_ deficiency

**DOI:** 10.1007/s10048-020-00624-3

**Published:** 2020-08-24

**Authors:** Matthias Amprosi, Michael Zech, Ruth Steiger, Wolfgang Nachbauer, Andreas Eigentler, Elke R. Gizewski, Michael Guger, Elisabetta Indelicato, Sylvia Boesch

**Affiliations:** 1 Center for Rare Neurological Diseases, Department of Neurology, Medical University of Innsbruck, Innsbruck, Austria; 2 Institut für Neurogenomik, Helmholtz Zentrum München, Oberschleißheim, Munich, Germany; 3 Neuroimaging Research Core Facility, Medical University of Innsbruck, Innsbruck, Austria; 4 Department of Neuroradiology, Medical University of Innsbruck, Innsbruck, Austria; 5 Clinic for Neurology 2, Kepler University Hospital GmbH, Linz, Austria

**Keywords:** Ataxia, Dystonia, Coenzyme Q_10_, Magnetic resonance spectroscopy, Ubiquinone, Mitochondrial disease

## Abstract

The spectrum of coenzyme Q_10_ (CoQ_10_) deficiency syndromes comprises a variety of disorders, including a form of autosomal recessive cerebellar ataxia (ARCA2) caused by mutations in the AarF domain–containing kinase 3 gene (*ADCK3*). Due to the potential response to CoQ_10_ supplementation, a timely diagnosis is crucial. Herein, we describe two siblings with a novel homozygous *ADCK3* variant and an unusual presentation consisting of isolated writer’s cramp with adult-onset. Cerebellar ataxia developed later in the disease course and remained stable during the follow-up. This report highlights that ARCA2 should be considered in the differential diagnosis of familial writer’s cramp.

**Electronic supplementary material:**

The online version of this article (10.1007/s10048-020-00624-3) contains supplementary material, which is available to authorized users.

## Introduction

Inborn errors of CoQ_10_ metabolism are an extremely rare and heterogeneous group of disorders which commonly present during childhood. They are caused by mutations in genes involved in CoQ_10_ synthesis [1]. Depending on the phenotype, patients may show a sustained improvement upon supplementation of CoQ_10_ and thus a timely diagnosis is crucial.

Autosomal recessive cerebellar ataxia 2 (ARCA2) is caused by biallelic mutations in the AarF domain–containing kinase 3 (*ADCK3*) gene, which encodes a mitochondrial protein essential in CoQ_10_ synthesis [2, 3]. Symptoms are variable, ranging from cerebellar ataxia to severe phenotypes including cognitive impairment, myoclonus, epilepsy and stroke-like lesions (ataxia in up to 100%, epilepsy in 40%, dysarthria in 34%, pyramidal signs in 32%, oculomotor dysfunction in 30%, tremor in 28%, dystonia in 21%, exercise intolerance in 17%, myoclonus and pes cavus/neuropathy in 13% [3]). Typical onset of ARCA2 is in childhood [4, 5]. The mean age of onset in one recent review, containing clinical data of 29 patients, was 5 years (ranging from 1 to 27) [6]. Up to 50% of ARCA2 patients benefit from oral CoQ_10_ supplementation [7]. In the literature, daily doses between 10 and 15 mg/kg of bodyweight up to a total of 2400 mg per day are recommended [1]. In addition, a trial duration of at least 6 months is advocated in order to detect a possible benefit [7].

Here, we report a novel *ADCK3* mutation, associated with an atypical presentation of neurological symptoms, comprising task-specific dystonia and ataxia, and adult-onset in one patient. Our report highlights the phenotypic variability of ARCA2 and its status as a rare but noteworthy differential diagnosis in adult patients with a suggestive combination of movement disorders.

## Materials and methods

### Clinical assessment

Extensive diagnostic work-up was performed consisting of neurological examination, neuropsychological testing, laboratory examinations, neurophysiological investigations and 3 Tesla cerebral magnetic resonance imaging (MRI), including proton (H^1^) and phosphorous (^31^P) spectroscopy. Part of the neuropsychological testing were the Wechsler memory scale, the Rey complex figure test, the “Verbaler Lern- und Merkfähigkeitstest” (a German version of the Rey’s Auditory Verbal Learning Test), the “Regensburger Wortflüssigkeitstest” (testing verbal fluency) and the Hospital Anxiety and Depression Scale. Up to four follow-up examinations, documented by the validated rating scale “Scale for the assessment and rating of ataxia” (SARA), are available (see Table 1) [8]. All investigations were performed in accordance with the Declaration of Helsinki. Both patients gave written informed consent for genetic testing and publication.

**Table 1:** Demographics and clinical presentation

Patient	Gender	Age at onset	Age at first examination	SARA score							Cerebellar syndrome	Writer’s cramp
				Years of follow-up								
				0	1	2	3	4	5	6		
III-1	♀	20	45	13	13	-	-	13	13	13	Yes	Yes
III-3	♂	7	28	10	10	11					Yes	Yes

### Whole exome sequencing

DNA fragments were enriched with a SureSelect Human All Exon Kit (Agilent, 50 Mb V5) and subsequently sequenced on a HiSeq2500 (Illumina) to an average coverage rate of more than 140. More than 98% of target sequences were covered at least 20 times [9].

## Results

Family history revealed two out of three siblings were affected. The family originates from a rural area in Austria (Fig. 2a). The first affected sibling (index patient, III-1 in the pedigree), 50 years old, is 20 years older than her affected brother (III-3 in the pedigree), 30 years old. The index patient developed symptoms at the age of 20, around the time her affected brother was born. Additionally, family history showed a high prevalence of tumours (lung cancer in the paternal grandmother and grandfather, leukaemia in one paternal uncle, unspecified malignancy in one paternal and one maternal aunt) but did not reveal any movement disorders. At the time of the patients’ last visit, the father (II-4 in the pedigree) was 73 and the mother (II-5 in the pedigree) 69 years old.

### Index patient

**Fig. 1:**
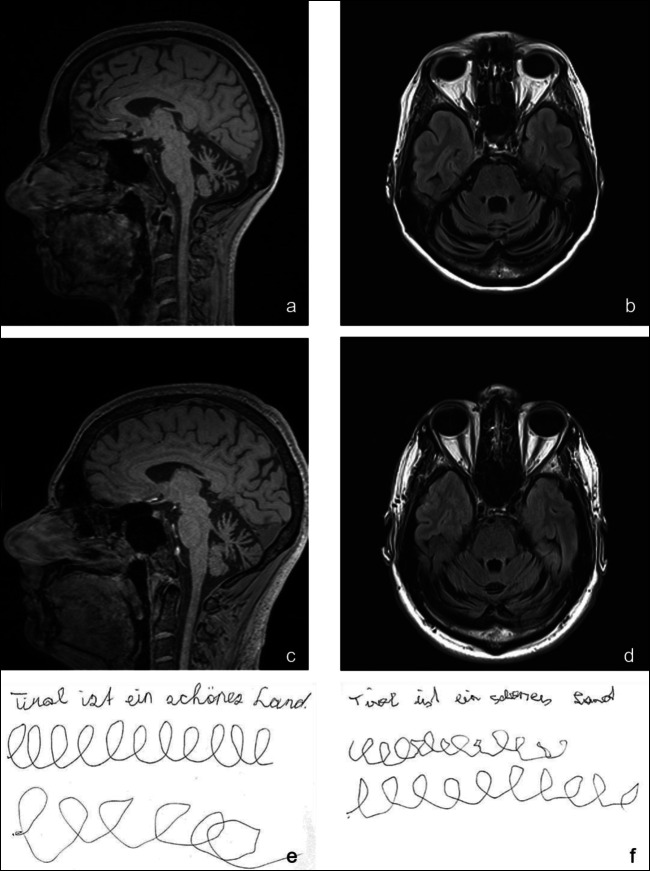
Sagittal and axial MRI imaging demonstrating cerebellar atrophy in the index patient (a and b) and patient III-3 (c and d). Writing samples depicting writer’s cramp associated dysgraphia (full sentence and first L-letter series written with dominant hand, second L-letter series drawn with non-dominant hand) in the index patient (e) and patient III-3 (f)


The index patient had normal development and uneventful clinical history until the age of 20. She then experienced tremulous dystonic posture when writing (Fig. 1e), meeting the diagnostic criteria of writer’s cramp. The patient’s symptoms remained unchanged until the age of 34, when she experienced balance problems while riding a bike. Around the age of 41, she recognized progressive unsteadiness of gait and dysarthria. On examination at the age of 45, she displayed writer’s cramp and cerebellar ataxia (Video Supplement 1). After a disease duration of about 5 years, she presented with a SARA score of 13 out of 40 points (Table 1). Neuropsychological testing revealed an average to above-average performance in cognitive tasks such as attention, memory, frontal executive functions, verbal fluency, spatial performance and depression and anxiety scores. Nerve conduction studies showed no signs of polyneuropathy. Extensive laboratory work-up, including serum lactate, alpha-fetoprotein, serum-copper and ceruloplasmin as well as vitamins B1, B6, B9, B12, A, E and folate yielded normal findings.

ESM 1(MP4 107,250 kb)

Follow-up visits showed stable disease, with SARA scores unchanged and task-specific dystonia still present. It is notable that due to the mild symptoms, the patient is still able to work as a physiotherapist.

### Patient III-3

The younger brother of the index patient had a normal early development until the age of seven, when he started to experience writing problems (Fig. 1f). In contrast to his older sister, he noticed some imbalance of gait and slurred speech before the age of 10 years. Besides some clumsiness in sports, the patient experienced no relevant impairment in daily life. At first visit in our ataxia clinic, this 28-year-old patient presented with a cerebellar syndrome. Like his sister, he displayed writer’s cramp (Video Supplement 2). Cognitive functions were unremarkable. Initial SARA score was 10 points and remained stable over follow-up. Evoked potentials, nerve conduction studies and laboratory parameters were within normal limits.

ESM 2(MP4 92,531 kb)

### Genetic testing

**Fig. 2:**
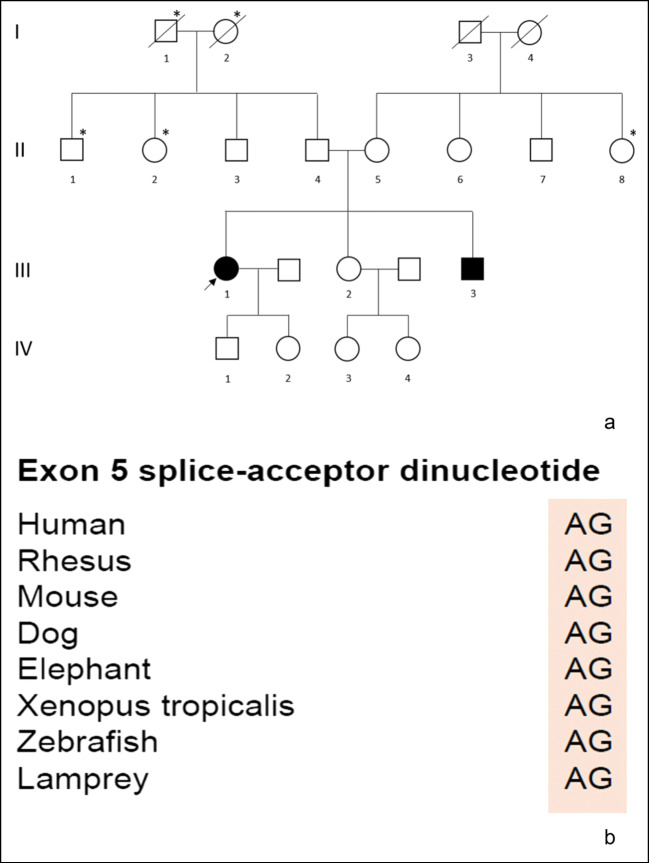
(a) Pedigree of the family with index patient indicated by an arrow. Roman numbers: generations; Arabic numbers: individuals within each generation; circles: women; squares: men; diagonal line: deceased subjects; black: individuals with ARCA2; asterisk: malignancy. (b) Conservation of the described variant among other species

Genetic analysis of repeat expansion disorders, specifically spinocerebellar ataxia (SCA) 1, 2, 3, 6, 17 and Friedreich’s ataxia, was negative. Whole exome sequencing (WES) revealed a novel splice-site mutation in the *ADCK3* gene on chromosome 1 which was not found in a large in-house control exome dataset (Helmholtz Center Munich) and around 135,000 individuals of the Genome Aggregation Database (gnomAD) [10]. Both patients were homozygous for a canonical splice-site variant c.656-1G>T (accession number: NM_020247.4), which is predicted to cause aberrant splicing. The variant is located at the intron 4-exon 5 boundary (exon 5 splice acceptor loss) and the deleteriousness prediction Combined Annotation Dependent Depletion score was 23.2 (variant predicted to be among the top 1% of most deleterious variants in the human genome: https://www.ncbi.nlm.nih.gov/pmc/articles/PMC3992975/). The variant was classified as “pathogenic” according to the guidelines of the American College of Medical Genetics and Genomics [11] and is in a highly conserved region (Fig. 2b).

### Coenzyme Q_10_ supplementation

After diagnostic confirmation, both patients received daily oral supplementation with 60 mg of ubiquinol, the chemically reduced form of CoQ_10_. Due to lack of benefit, the index patient decided to stop the supplement after 2 months. Her brother stopped ubiquinol because of frequent headaches and switched to CoQ_10_ therapy for 1 year. He is still taking an increased dose of 600 mg CoQ_10_ daily_._

### Magnetic resonance imaging and magnetic resonance spectroscopy

The index patient’s MR imaging showed cerebellar atrophy, most prominent of the superior vermis and superior cerebellar peduncles, along with a small temporal lesion from a subacute stroke she suffered at the age of 44. MR imaging in her younger brother revealed symmetrical cerebellar atrophy, predominantly of the upper vermis and superior cerebellar peduncles (Fig. 1a to d). ^31^P-MR spectroscopy was performed in both siblings in order to detect possible energy deficits in the phosphocreatine pathway and showed findings within normal range. In H^1^-MR spectroscopy, no lactate peaks were observed.

## Discussion

Here, we describe a novel homozygous *ADCK3* mutation in a sib-pair of Austrian origin with a noticeably mild phenotype, consisting of writer’s cramp and cerebellar signs with adult-onset.

Up to date, 57 cases of *ADCK3*-related ataxia with heterogeneous presentation have been reported in the literature [3]. The spectrum of reported manifestations includes ataxia, seizures, pyramidal signs and cognitive impairment. Ataxia is a constant feature and usually the first symptom at disease onset. Dystonia, particularly writer’s cramp, has been also reported, but it usually manifests later in the course of the disease [3]. Deep phenotyping in our patients, however, revealed dystonia as a manifesting symptom. Moreover, we found only six cases with adult-onset in the literature. Like our patients, adult-onset ARCA2 cases exhibited a milder and slowly progressive disease [3, 5, 12–15].

Genotype-phenotype analysis of a small series in the literature did not find relevant phenotypic difference between cases with nonsense or missense variants [3]. Notably, clinical presentation may differ markedly within the same family. In a report by Blumkin et al., two siblings sharing another compound heterozygous mutation (p.P502R and p. Thr584delACC) had a similar age at disease onset, but presented with highly variable clinical phenotypes (childhood onset of mild dysfluency and clumsiness without progression in one sibling and a progressive generalized cerebellar syndrome combined with cognitive impairment in the other) [16]. On the contrary, the affected siblings in this report displayed a similar phenotype, but with a marked difference in the age at onset.

CoQ_10_ supplementation was started around 20 and 25 years after disease onset. Supplementation did not result in a subjectively relevant benefit. Since there was no disease progression during a 6- and 2-year follow-up period, as captured by stable SARA scores, it remains speculative whether a prompt supplementation with CoQ_10_ would have prevented progression of ataxic symptoms. In the literature, a minimal supplementation period of 6 months has been recommended [7]. In this study, however, CoQ_10_ supplementation in the index patient was stopped after only 2 months due to incompliance. Together with the low starting dosage, this could be one possible reason for the subjective lack of benefit in patient 1. In her sibling, dosage of CoQ_10_ was increased to 600 mg daily, corresponding to around 5 mg/kg bodyweight. Due to reported adverse event upon a previous trial with ubiquinol, a slow increase of CoQ_10_ dosage is planned for the next follow-ups. SARA scores and subjective reports from patient III-3 did neither reveal improvement nor disease progression.

One limitation of this study is that no biochemical analysis of CoQ_10_ in muscle cells or fibroblasts was performed. Analysis of patient-derived cells could be used to validate the pathogenicity of the mutation. Because of clear pathogenicity of the detected ADCK3 variant and presence of a concordant phenotype, biochemical and molecular analyses in a muscle biopsy were not required to confirm the diagnosis in this case. Furthermore, genetic testing in the parents has not been done as they were not able to attend to an in-person visit at the clinic due to the distance from our centre and additional personal reasons.

Several reviews addressed the issue of differential diagnostics in the setting of autosomal recessive ataxia [17–19]. After having excluded the most frequent causes of inherited ataxia (namely Friedreich’s ataxia and the polyglutamine SCAs), clinical features, findings from ancillary examinations and therapeutic consideration guide the further diagnostic steps. In the presence of a phenotype with cerebellar ataxia and dystonia, especially treatable disorders such as Wilson’s disease, cerebrotendinous xanthomatosis, Niemann-Pick type C, abetalipoproteinemia, GLUT1-deficiency and ataxia with vitamin E deficiency should be ruled out [20]. Eventually, the application of whole exome sequencing can enable the detection of extremely rare variants which also bear relevant therapeutic consequences, as in the present case.

Taken together, our findings show that the combination of writer’s cramp and ataxia, irrespective of age at onset and the chronological occurrence of symptoms, could be indicative of ARCA2. In particular, our report suggests *ADCK3* variants should be considered in the presence of familial clustering of writer’s cramp. In a previous study, “writing difficulty” was described as an early finding in 13% of patients, although the dystonic nature of this impairment was not specified. The combination of writer’s cramp and mild ataxia has only been described in 10 patients so far. Apart from one patient, however, all showed first signs of disease between the age of 2 and 15. A literature review by the same authors did not show dystonia or writer’s cramp as first symptom in any adult-onset case [3]. Awareness of atypical phenotypes is therefore crucial to prompt earlier diagnosis and therapy. Further reports are advocated to define the course of the disease upon an early CoQ_10_ supplementation.

## Data Availability

Detailed genetic and clinical data are available upon request.
